# Researches on the Development, Structure, and Diseases of the Teeth

**Published:** 1839

**Authors:** 


### RESEARCHES ON THE DEVELOPMENT, STRUCTURE, AND DISEASES OF THE TEETH. By *Alexander Nasmyth*, F.L.S. F.G.S. &c. Member of the Royal College of Surgeons, London, &c. 8vo. pp. 165, London, 1839.

WE need not, at this time of day, indulge in a disquisition on the uses of the teeth, nor on the importance, not to say necessity, of being acquainted with their structure, in order to understand thoroughly their diseases.

Mr. Nasmyth, the author of the work before us, is a gentleman much esteemed by those who have the pleasure of knowing him, and distinguished for the zeal and ability with which he cultivates dental surgery. The volume before us is but the commencement of the work which Mr. Nasmyth promises, and of which this bears the title. We have here only the introduction to the whole—the part which contains the anatomy and physiology of these organs.

It may seem surprising to such of our readers as are out of the pale of the anatomical and physiological inquiries of the day, that 165 pages of close letter-press should be dedicated at the present time to such a subject. But the researches of minute anatomy are endless. While fresh enquirers are to be got, fresh discoveries will assuredly be made. The microscope has the brilliant advantage of allowing the most discursive ranges of fancy, and *its* field has a boundless horizon, The microscopist of France sees something very different from what is visible to the ken of him of England, and the German porer upon glasses looks, of course, much farther than either. The only standard that we can conceive able to settle disputes, is an Act of Parliament, and the attention of the Legislature ought really to be called to it. Lecturers and Journalists are much to be pitied. They *get up* some piece of minute anatomy one season, the last, the finest, and the best. But before the next year arrives, some new microscope or microscopist has come out, the last discovery is pronounced mere moonshine, and the fresh one is to be rehearsed, and performed to the gaping audience, with the same fate before it as overtook its predecessor. This is too bad.

"The Historical Introduction," observes Mr. Nasmyth, "with which the present work commences, occupies a much larger space than I at first intended to allot to it: its length is owing to the variety and importance of recent investigations in this department of science, conducted principally by Swedish and German anatomists. It still, however, might have been enlarged in that part which treats of old writers; but I have been induced to omit many of their statements, as being more curious and fantastic than practically valuable: the labours of many modern writers of ability might also be added to it, but not with much advantage. From the very commencement of it the reader will be struck with the conflicting nature of the evidence which it contains on even the most elementary points. I am inclined to think, however, that these discrepancies have not their origin so much in absolute error, or in defect of observation, as in the constantly varying condition of the parts which have been described, and in the diversity and imperfection of the means which have been used for their examination. Without recurring to such an explanation as this, it is impossible to conceive how such men as Hunter, Cuvier, Bichat, and Blandin, for instance, can often flatly contradict each other. Those who have hitherto published the results of their experiments in this department of anatomy, have, for the most part, omitted to give an account of their methods of investigation, and of the species, age, &c. of the subjects which have been submitted to examination; thus rendering it impossible to test the accuracy of their facts, and to discover the source of fallacy either in the new views, or in those which had hitherto been current.

I have endeavoured to conduct my investigations on a scale which I conceived commensurate with the importance of the subject; and in detailing the results which I have arrived at, I shall give as careful a description as possible of the nature of the experiments, dissections, or observations which led me to them. The substance of what I have to offer will go to prove, I think, a greater degree of simplicity and uniformity, than has hitherto been suspected in the organization of these structures." *Pref.* x.

Mr. Nasmyth proceeds to remark on the wonders elicited or created by the microscope, in modern hands, and he adds:—

"In 1837, I learnt that Professor Retzius of Stockholm had published a very interesting series of microscopic investigations on the structure of the teeth, and I then made every exertion to obtain his publication, but without success. I first saw it in April 1838, when I was favoured with a loan of it, by my learned friend, Dr. Grant.

The interest excited by the new views of this intelligent and industrious Swedish physiologist, induced me to resume a course of investigations on all the points connected with the structure of those organs, and I immediately announced my intention of publishing on the subject, and pursued my investigations with assiduity. Not less than five years ago, I made many observations on the structure of the teeth, and saw at that time their striated appearance; but being unable satisfactorily to explain this, and knowing that it had already been recorded by Cuvier, I did not attach sufficient importance to it, as it did not then seem to me to be likely to lead to any practical results. My observations were shortly afterwards interrupted, owing to the imperfections of the microscope which I then used, and to the difficulty I experienced in making suitable sections of the teeth.

I did not at first intend to publish the whole of Retzius's account of the tubular system in different animals, but I at length determined to do so, as I found that it could not be divided without injury to the whole, and that, moreover, I should frequently be compelled to refer to almost every part of it.

Those who contemplate the teeth merely as ornaments, or consider them in no other light than as the active agents at a feeding trough, or as the formidable weapons of the wild tenants of the forest, will, doubtless, regard the details of Retzius as irksome and tedious; but he who with ardour and admiration tracks the steps of Nature through the regions of Zoology, will welcome with delight a full description of these new researches; he will recognise at once their importance, and cannot fail to be struck with the unostentatious manner in which a great mind has opened to the world a new field of exertion, foreseeing its value to the student of Geology as well as of Natural History. But I have been still further induced to publish the researches of Retzius, from having myself made numerous observations and experiments on the same subjects, which I hope will be found to illustrate some of them, and to carry out others to very interesting and original conclusions.

The importance of the study of Odontology in a zoological and geological point of view, has induced me to form as complete a collection as possible of microscopic preparations of the teeth of the various classes, both living and extinct, of the animal kingdom. From these I intend, in the course of the present work, when treating of the structure of the teeth, to make a selection calculated to serve as an index of the type of any animal. This part of the work I think will prove of much use to the Geologist, in enabling him to state to what class of animal any tooth, or fragment of tooth, belongs." xiii.

Our readers are in possession of the objects of Mr. Nasmyth, and may guess our own, in introducing him. A short time back, we presented a pretty copious account of the work of M. Blandin on the Anatomy and Physiology of the Teeth, and in bringing our account of both up to Mr. Nasmyth and the present time, we but redeem our pledge of keeping pace with information, in its advancing or its dodging march. We shall endeavour to combine precision and brevity in our notice of the copious pages before us.

We may remark more particularly, that this is but the "historical" Introduction, that is, presents us only with an account of what Mr. Nasmyth's predecessors or contemporaries have effected. It is reserved for future Parts, to inform us of the "development and structure" of the teeth, and to offer, we presume, the digested labours of Mr. Nasmyth himself.

We know not how we can better exhibit *in petto*, the plan, and arrangement, and, we might almost say, the contents of the work, than by quoting the author's own tabular view of them. Its copiousness will hardly be disputed. Here it is:—

Mention made by HERODOTUS of Egyptian dentists—Views of HIPPOCRATES on the structure of the teeth—Opinions of ARISTOTLE, of ARETÆUS,—of PLINY—Progress made by GALEN, who describes these organs as bones—Details on this subject by AETIUS, RHAZES and ABULCASIS—Writings of VESALIUS and EUSTACHIUS—Doctrines of AMBROSE PARÉ, of SCALIGER and KERKRING—New views promulgated by MALPIGHI, and comments upon them by RETZIUS—LEEUWENHOEK'S announcement of the discovery of tubes in the dental bone—MALPIGHI on the fibres of the enamel—DUVERNEY on the developement of the teeth—WINSLOW on the enamel—BERTIN—HERISSANT'S paper on the formation of the enamel, and on the organisation of the gums—JOURDAIN on dental developement—Opinions of HUNTER, physiological, pathological, and practical: singular resemblance on some points between him and AMBROSE PARÉ.

Commencement of the modern anatomical era—BICHAT on the teeth—BLANDIN. Works which have been published on the teeth in Great Britain during the present century—Essay of Dr. BLAKE—Work of Mr. FOX—of Mr. BELL, &c. Consideration of the French school resumed—G. CUVIER,

"Sur les ossemens fossiles"—Serres' views—F. Cuvier's work on the Teeth of the Mammalia—Rousseau's Comparative Anatomy of the Dental System. Modern German writers on this subject—Weber in Hildebrand's Anatomy—Schreger and Wagner. Views of various writers on the structure of the ivory—G. Cuvier on the enamel—Hunter, Tenon, and Cuvier on the *crusta petrosa*.

Recent researches on the structure of the teeth—Purkinje and Müller on the tubes in the dental bone and on the fibres of the enamel—Retzius' microscopical researches on the structure of the ivory, enamel, and crusta petrosa in the teeth of mammalia, reptiles, and fishes—His summary and physiological conclusions—Mr. Owen's paper read at the British Association.

Recent researches on the developement of the teeth—Mr. Goodsir's paper in the Edinburgh Medical and Surgical Journal—Arnold on the dental follicle—Original and historical details of Valentin—Raschkow on the origin of the dental follicle—on the enamel-organ—on the dental germ—on the formation of the enamel—on the cement—on the alveolar membrane—on the developement of the gum.

Retrospective review and summary of the present state of information. State of the questions concerning the origin of the dental follicle—concerning the capsule itself—the osseous nature of the teeth—their vascularity—their composition and structure. Reflections on the evidence adduced in favour of the existence of a tubular system—Erroneous views of Müller on the crusta petrosa—On the admission of a fourth substance as entering into the composition of the teeth—Concluding remarks on the methods of conducting experiments on these organs.

Our main intention being to lay before our readers the results of the more recent and esteemed investigations, we shall proceed at once to Purkinje, Müller, and Retzius.

### 1. Structure of the Teeth.

A. *Purkinje's and Müller's Observations.*—The researches of Purkinje have been given to the world, in a dissertation by one of his pupils, V. Fraenkel.

*The Ivory.*—Purkinje states that the substance of the tooth and the enamel have a near similarity to the structure of bone, but on the outer surface of the root, he says, that there is a layer of true osseous substance, in which, at any rate, may be seen the osseous corpuscula which he has discovered.* This same structure he has discovered in the cement of the teeth of several animals. The proper dental substance examined in thin polished lamellæ consists of a uniform structureless (*structurlos*) substance, and of fibres passing through it. These latter run in parallel lines from the outer to the inner surface of the tooth, obliquely in some places, straight in others: they are in every part nearly of the same size, and communicate by but few

* Müller states that he has had frequent opportunities of confirming these results.

branches with each other. Though they are very thickly placed, still the structureless intermediate substance forms the greater part of the mass of the tooth: as their diameter he gives one-fifth or one-sixth of the space between two of them. He has also discovered that these fibres are tubular, and that in the teeth of the horse, at any rate, they are capable of absorbing ink by capillary attraction—a fact which is also confirmed by Müller. But Purkinje does not pretend to pronounce on the contents of the tubuli. Müller helps him out.

The latter affirms that they, or parts of them are filled with inorganic, calcareous salts. He says that when the light falls on fine polished lamellæ of dental bone, the white colour of the tooth is soon seen to be owing to these tubes or fibres, and that the intermediate substance is more transparent; moreover, that when such sections are submitted to the action of an acid, the white colour of the fibres disappears, the remaining cartilage still presenting tubes in their interior, which, however, when dried, are no longer white. He saw in healthy as well as in carious teeth, that in parts of these tubes something was contained.

Linderer, continues Mr. Nasmyth, who has long been engaged in researches on healthy and decayed teeth, has observed, that the dental substance in the latter (even where the enamel only is affected) loses its colour in several parts between the carious spot and the internal cavity. As this is nearly always the case, it is evident that an alteration in the substance of the tooth must be produced by the superficial caries. In fine polished sections of such teeth, Müller could easily see with the microscope, that where they were become transparent, a crumbly substance was contained in parts of the tubuli, and that this substance was more coherent in the tubuli of the white tracts; by adding diluted acid, he also observed under the microscope that this crumbly substance was dissolved. He has often performed this experiment with the same result on fine polished slices of healthy teeth. In these he found individual fibres, frequently in considerable number, on which were dark spots following close on each other.

The result of these and some other observations is, *that* the tubes have an organised basis, a membrane, and that this in the solid tooth is stiff and brittle, and probably saturated with calcareous salts, but weak and soft in the tooth which has been deprived of the latter—and *that* the calcareous salts exist not merely in the tubuli, but also in the intermediate substance, which contains, at all events, the greater part of the calcareous earth, either chemically united with the cartilage, or deposited in it in an invisible manner.

The interior of the roots of old teeth consists, according to Purkinje, not of dental substance, but of bone properly so called, as does also the deposit on the external surface of the root, and this statement is confirmed by Müller.

Our readers are aware that many anatomists class the teeth with horny rather than with bony productions, and consider them as growths from the tegumentary system. But though they grow in layers like horn, still they contain none, as Müller has repeatedly satisfied himself, but true cartilage yielding glue. From the dental cartilage of a horse's tooth, he obtained by boiling a large quantity of beautiful colourless glue. Whalebone, and other corneous formations, which supply the place of teeth, consist, he has found, of true horn, and furnish no glue, however long they may be boiled. It would seem from this, that horn supplies the place of dental cartilage when the teeth contain no deposition of calcareous earth. This is the case, at any rate, with the vertebrata. It seems to us that teeth hold an intermediate place between true bone and horn. They have analogies with either, closer to the former nearly in proportion to the superiority, to the latter nearly in proportion to the inferiority of the animal in the zoological scale.

*The Enamel.*—According to Purkinje's researches, continues Mr. Nasmyth, the enamel consists of simple perpendicularly-placed fibres, increasing somewhat in size towards their upper extremity, forming quadrilateral prisms, and often making several curves on one and the same level. These fibres are adherent to lamellæ, which run transversely round the tooth, and generally rest obliquely on the surface of the dental substance. These observations are fully confirmed by Müller, who has examined the last molar tooth of a calf, on which the enamel was still soft, and found that even in this state the latter consists of separate prisms, as yet uncemented, but held together by a transparent fluid matter. These firm but somewhat flexible fibres are not easily acted on by acetic acid, but quickly dissolve in muriatic acid, without any perceptible escape of air.

B. *Observations of Retzius.*—These observations are so voluminous or so minute, that it is difficult to know where to begin or stop, so as to suit the readers of a journal. Passing over between sixty and seventy pages, chiefly occupied with the minute anatomy of teeth in various animals, we arrive at the conclusions which Retzius draws from his own researches and observations, and we shall endeavour, so far as is practicable, to condense his summary. Analysis is next to impracticable, and abbreviation must principally consist in omission. We may premise what we shall insert by the remark, that Retzius owns and regrets the imperfection of his investigations and his knowledge. We proceed with Retzius' summary.

"The teeth of mammalia consist in general of three substances, the dental bone, the enamel, and the cortical substance. These three substances form also the teeth in certain amphibia and fishes. The dental bone contains tubes and cells which are in communication with each other, and which are analogous to the minute tubes and cells forming an important part of the organisation of bone.

The minute tubes in the osseous substance I demonstrated, together with those of the dental bone, by making slices of teeth which were still fixed in the alveoli. Preparations made in this manner contained of course a portion of the alveolar bone, which, when it was filed as thin as the sections of the tooth, illustrated the internal structure of the dental bone. These tubes in bone itself had been shortly before found by Müller and Miescher, as also by Purkinje and Valentin. Müller was the first who showed that they contained calcareous matter. They are still more minute in bone than in the tooth, and are many times smaller than the most minute blood-vessels, their thickness, according to Müller, being $\frac{1}{5000}$ line P. M."

"They radiate from the minute medullary canals, which were first more carefully examined by Purkinje, give off an abundance of branches which form reticular communications, and terminate in Purkinje's corpuscula, or cells. I call them by the latter name, because I am convinced that they are excavations in the substance of cartilage as well as in that of bone, which partly contain a clear fluid, and partly deposits of calcareous salts." 114.

" The tubes in the dental bone open into the cavitas pulpæ, and give off branches in their course, which form a network of communication between each other, and which terminate in cells. These cells, together with the branches from the main tubes, nearly fill up the interstices between the latter. The breadth of the main tubes varies from $\frac{1}{400}$ to $\frac{1}{1000}$ of a line, P.M., and diminishes as they divide into branches: the most minute subdivisions are many times smaller than $\frac{1}{1000}$ of a P. line. It is very likely that the tubes and cells which are seen under the microscope form but a small portion of those which really exist.

The dental bone, as Cuvier and several others have described, is deposited layer by layer round the surface of the pulp, and the most external layer is that which is first deposited. During this process the most external closely contiguous cells have their origin, and also the peripheral extremities of the main tubes. During the formation of successive layers, the latter are also continued inwards; and it would seem as if their parallel curvatures were produced during their continuation from one layer into another. In this case we have grounds for presuming that there is a periodical motion of the pulp, by means of which the tubes, whilst in process of formation, are at one time pushed towards the apex of the tooth, and at another drawn down and in the direction of its root. We may also imagine the periodical motion to be of various kinds. The most delicate and most numerous undulating curvatures must be the work of very short periods. The longer, shallower curves, of which every fully developed tube presents only a few, may be regarded as marks of other and slower periodical changes in the formation of the pulp, during which those producing the short tubular undulations are carried on uninterruptedly." 115.

These processes, however, continues Retzius, are only carried on with complete regularity before the tooth protrudes through the gums. In the extremities of the roots, and in the osseous mass filling up the centre of the cavitas pulpæ, which are subsequently formed, the tubes are less regular and uniform than in other parts. In this last formed osseous mass, the branches of the tubes are best shewn, because both the former and the latter have no longer their parallel arrangement. In this irregular form of dental bone the largest cells are found, and the entrance of the tubes into the latter is admirably well seen, as also the reticular meshes formed by the tubes themselves. In many places this substance presents so great a similarity to other osseous formations, that it appears rather to belong to ordinary bone than to dental bone; it differs, however, from the former in the larger size of its main tubes.

After some remarks, the gist of which is to shew, *that* the similarity between dental bone and real bone is greater than, at first sight, it would seem to be—and *that* the main difference consists in the manner of their formation, the external layer being the first formed in dental bone and the last in real bone—Retzius goes on to state that, it can scarcely be doubted that the minute tubes in dental bone, in the cortical substance, in common bone, and in the horns of deer, as well as the cells which are united with them, are a peculiar kind of vessels, containing a nourishing and supporting fluid, of which probably the composition differs at different periods. This fluid is most likely secreted by the capillary vessels which clothe the surface of the pulp.

Retzius adverts to the subject of the vital processes going on in teeth—a difficult matter, on which we confess that he does not much enlighten us; in fact, he leaves the difficulty as he found it. His idea would appear to be, that the solid matter of the tooth, once formed, is not changed, but that there exists a renovating circulation of the dental fluids in the minute tubes and cells.

"The *enamel* presents a much more simple structure, being without sanguiferous or any especial vessels: its structure most resembles that of the crystalline lens. It also probably requires an organic fluid for its preservation, which, as I presume, is conducted to it by the tubes of the dental bone, permeating afterwards the membranous parietes which probably clothe the individual fibres. This substance is not found in all teeth, but appears in many cases in a rudimentary state, so to say, inasmuch as it is missed in the teeth which have not yet or have only just protruded—as in the anarrhichas lupus, gadus molva, and phoca annellata." 121.

The *cortical substance,** continues Retzius, is found on the teeth of most mammalia, and is also met with on those of amphibia and fishes. It is everywhere distinguished by a predominant abundance of osseous cells, and by less extended, in general more minute, and often altogether irregular osseous tubes. It is formed in some animals (in the elephant, the horse, the ox, &c.) as the outer covering of the enamel, particularly within the folliculus dentis before it is closed; but in these animals also it is secreted during the whole of life from the membrane surrounding that part of the tooth which is concealed in the alveolus. In most animals with simple teeth, as also in man, it only invests that part of the tooth which is not covered with enamel. In the bradypus, in which no enamel is found, it covers the whole tooth. Its deposition round the root of the tooth increases as the cavitas pulpæ closes, and as the pulp itself correspondingly diminishes. The minute osseous tubes in the cortical substance then form immediate communications with the osseous cells and osseous tubes in the dental bone, so that the latter can now, when the pulp has as good as ceased to exist, receive the necessary supply of fluids from without. In certain animals the cortical substance presents also larger canals, which are of about the same thickness as the medullary canals in bone. In the cortical substance closing in some teeth the end of the cavity of the pulp which would else be open, those tubes open into the said cavity, and contain the blood-vessels which belong to the pulp itself. The larger tubes which are found in that portion of the cortical substance lying exposed beyond the gum, appeared empty, and are probably only the remains of the blood-vessels which were engaged in its formation. It is, however, still probable that even in this substance a circulation is kept up of peculiar fluids secreted from the blood, which fluids rise into the peculiar minute tubes and cells, in much the same manner as the sap rises in a plant.

The layers of dental bone are deposited, as above described, from without inwards, the most external being thus always the first formed: in the cortical substance the reverse obtains,—its inner layers contiguous to the surface of the tooth are first formed, and afterwards the outer ones.

The cortical substance presents no similarity to tartar; and one of the best proofs that the latter is a mere deposit from the saliva, is the fact that we find thick crusts of it round artificial teeth.

Such are the chief facts determined by Purkinje, Müller, and Retzius.

* The "crusta petrosa," as it is usually denominated.—*Rev.*

Mr. Owen has re-investigated the subject, and lent to their views the sanction of his concurrence. And he has arrived at a conclusion, previously entertained by Mr. Nasmyth, *that* the structure of the teeth, as manifested by means of the microscope, forms a new, distinct, and specific guide for classifying the different members of the animal kingdom, and determining their respective types. From the enduring nature of these organs, the characteristic modifications which they present, will form, as Mr. Owen has admirably pointed out, a most valuable accession to geological science.

Mr. Nasmyth devotes some thirty pages to the labours of Arnold, Raschkow, and Goodsir, on a subject of great moment—the *development of the teeth.* A subject, too, of difficulty almost proportioned to its magnitude. Everybody conversant with anatomy, everybody who has read, or studied, or worked in any way upon the teeth, is aware of the obscurities that beset the investigation of their development, and of the inconsistencies, theories, and contradictions of the physiologists who have meddled with it. Our readers may perhaps suppose that, with Arnold, Raschkow, and Goodsir, we turn over a new leaf—that at last microscopes have been found to tally—that something like the phenomenon of three dental anatomists not contradicting each other point-blank is to be witnessed. Simple readers, men of easy faith! Arnold, Raschkow, and Goodsir, are absolutely positive and negative poles in their statements.

"The department of anatomy under consideration," naïvely remarks our author, "has shared the fate common to almost every branch of the subject; that is to say, what has been affirmed in it by one inquirer has been systematically denied by the next. Raschkow and Purkinje directly and positively contradict the opinions of Arnold and Goodsir." 131.

Such being the case, we think we shall consult our readers' interests by allowing these gentlemen to arrive at something like unanimity, before we cite their facts or fancies.

But Mr. Nasmyth himself has hitherto scarcely appeared upon the page. He has been seen or heard but as the chronicler of others. It is in future parts that he will speak in propriâ personâ, and, from what we know of him, we are sure that he will speak well. Yet even in this he says a little. The last few pages are occupied with a retrospective glance at the historical sketch which has terminated, and contain a faint attempt to extract from it what our utilitarian practitioners love, some precept or lesson of practice.

1. After pointing out the questions (the easy labour) on which anatomists are all at loggerheads, Mr. Nasmyth arrives at one on which (observe the prodigy!) they are all agreed.

"There is one opinion which has been uniformly maintained by all writers on this subject, viz. that when the teeth of man, or other analogous simple teeth, are extruded, the enamel is divested of all external covering, and exposed immediately to the atmosphere, denuded of both the envelopes of capsule and mucous membrane which it possessed during what has been called the saccular stage; and this denudation has always been supposed to be effected by the disruption of these coverings. My own views, however, on this point, which are contained in a paper read some time ago before the Medical and Chirurgical Society, are different from those generally received; and I have no doubt that I shall be able to prove that it is a process of absorption, and not of disruption, by means of which the tooth is emancipated, and that when perfected and extruded it is still invested with a capsular covering. This covering, as it is seen to be continuous with the crusta petrosa, I consider to be analogous to it, as the latter has been found to invest the whole tooth in an enlarged state in many animals, and in a particular interesting form in the orycteropus, bradypus, walrus, &c." 154.

We *did* opine that enamel would have been left to us. But, alas! Mr. Nasmyth has given it a varnish of crusta petrosa. *Crusta petrosa* indeed!

2. It has been left, says Mr. Nasmyth, to the sagacity of Raschkow, one of the most recent writers in this department, to explain what Herissant calls the *temporary gum*. Other writers have presumed that the tooth, before its extrusion, has a cartilaginous investment, forming of course a considerable obstacle to its progress; but the truth is, that this covering, instead of being cartilaginous, is merely composed of thickened epithelium.

The minute and interesting bodies first noticed by Cloquet and Serres, and denominated by them the dental glands, have been denied to exist by some inquirers, and one writer has gone so far as to assert that they are "too manifestly a mistake to require any particular refutation." They certainly, however, do exist, though probably they are neither entitled to the appellation of glands, nor endowed with the permanent functions assigned to them by Serres and Blandin.

3. The alveolo-dentar periosteum costs Mr. Nasmyth the enumeration of some eight or nine opposed statements. Each author was probably as certain that he was right, as Mr. Nasmyth is that *he* is right.

"It appears to me to be certain, that the external periosteum sends a continuation to line the cavity of the alveolus simply, and that the fang of the tooth continues invested by the original capsule." 157.

4. On the structure of teeth, we may be amused, if not satisfied, with our last quotation:—

"Hippocrates taught that they were composed of *hardened fat*. Leeuwenhoek was the first who stated that their structure is *tubular*, and this doctrine, after having been forgotten for one hundred and sixty years, has lately re-appeared in a most attractive garb. Monro believed that these organs are *longitudinally fibrous*: Fox, that they were *deposited in layers*. G. Cuvier says, 'that they are *neither cellular* nor *fibrous*, but composed of *laminæ*.' Bichat held that they were analogous to *rock*, and *fibrous*; Serres, on the other hand, sees *nothing like fibres* in them. Rousseau describes their structure as being *longitudinally striated*. Blandin, who wrote so late as 1836, tells us, that they are composed of *plates*, situated parallel to their external surface; and this statement was made a year after Purkinje, and others, in Germany, had so beautifully demonstrated their fibrous texture. This latter subject has been further elucidated in the most complete manner by Professor Retzius; and that the structure of the ivory is at any rate fibrous, could not now be denied even by M. Serres, or by those who have stood up for its rocky or simply stratified character.

The labours of Retzius and his contemporaries must be considered as a most splendid contribution to minute anatomy; but I still think that the evidence in favour of the existence of a *system of ramifying tubes* in dental bone or ivory, requires much further examination and corroboration, before we can be justified in adopting a theory tending to overthrow many received opinions, results of ordinary experience. The difficulty in arriving at just views of this subject is partly owing, I think, to the circumstance that writers have comprehended very different structures under the same term. All the diversified textures entering into the composition of the *bodies* of different teeth have been described as dental bone or ivory; and it is unfortunate that the advocates of a tubular system have not been much more precise than their predecessors in defining the specific nature of the various dental structures, in which they state that they have traced hollow fibres.

Like all newly-propounded doctrines, the theory of a tubular system, as laid down in the preceding pages, will be found to present some internal contradictions. 'It can *scarcely be doubted*,' says Retzius, 'that the minute tubes in dental bone, in the cortical substance, in common bone, and in the horns of deer, as well as the cells with which they are in connexion, are a peculiar kind of vessels containing a nourishing and supporting fluid.' In another part of his work, however, he says, 'with respect to the contents of the canals, I, as well as Professor Müller, have found that they consist of an inorganic or earthy substance, which appears white when viewed on a dark ground, but which disappears when the preparation is placed in diluted muriatic acid. When the light fall into these canals, this matter is seen to be composed apparently of infinitely fine particles, adhering together in lumps. The greater or less number and distinctness of these lumps seem to depend on the degree in which the preparation has been penetrated by water, oil, or turpentine.' 'That the small osseous tubes and cells,' he afterwards adds, 'contain osseous earth, is seen from their whiteness.' And one of his followers states, that 'these tubes are chiefly rendered visible by means of their contents.' How a tube can be filled with osseous matter, and at the same time allow of the circulation of a 'nourishing and supporting fluid,' I cannot understand. It must not be forgotten, too, that Retzius allows 'that, in the tooth, no renovation of the material appears to take place.'" 161.

We must take our leave of Mr. Nasmyth. Let us again press it on the attention of our readers, that this is the Historical Introduction to his work. As an original thinker and actor he is not yet before us. When he comes, we are confident that he will not falsify the character which we have given of him.

But we candidly own that we look with apprehension on researches into the minute anatomy of teeth, or their development. We are sure that most plain men will give them up in despair. The only way of forming a satisfactory opinion, is to adopt the Cadhi's plan, and hear but one side. If two are allowed to speak, the case is hopeless.

It will be when Mr. Nasmyth arrives at the practical portion of his subject that he will meet with most attention—excite most interest. We promise the public that they will not experience disappointment, for a more zealous and intelligent gentleman than Mr. Nasmyth we do not know.

